# Assessment of Gene Flow to Wild Relatives and Nutritional Composition of Sugarcane in Brazil

**DOI:** 10.3389/fbioe.2020.00598

**Published:** 2020-06-19

**Authors:** Eduardo Andrade Bressan, Igor Araújo Santos de Carvalho, Maria Teresa Mendes Ribeiro Borges, Monalisa Sampaio Carneiro, Edson Ferreira da Silva, Rodrigo Gazaffi, Regina Tomoko Shirasuna, Vinícius Abreu, Rafael V. Popin, Antonio Figueira, Giancarlo Conde Xavier Oliveira

**Affiliations:** ^1^Evolution Laboratory, Department of Genetics, “Luiz de Queiroz” Agricultural College, University of São Paulo, Piracicaba, Brazil; ^2^Technological Analysis and Simulation Laboratory, Department of Agroindustrial Technology and Rural Socioeconomics, Center of Agricultural Sciences, Federal University of São Carlos, Araras, Brazil; ^3^Plant Biotechnology Laboratory, Department of Biotechnology, Vegetal and Animal Production, Center of Agricultural Sciences, Federal University of São Carlos, Araras, Brazil; ^4^Plant Breeding Laboratory, Biology Department, Federal Rural University of Pernambuco, Recife, Brazil; ^5^Department of Biotechnology, Vegetal and Animal Production, Center of Agricultural Sciences, Federal University of São Carlos, Araras, Brazil; ^6^Herbarium Curatorship Research Nucleus, Vascular Plants Research Center, Institute of Botany, São Paulo, Brazil; ^7^Laboratory of Cell and Molecular Biology, Center of Nuclear Energy in Agriculture, University of São Paulo, Piracicaba, Brazil; ^8^Plant Breeding Laboratory, Center of Nuclear Energy in Agriculture, University of São Paulo, Piracicaba, Brazil

**Keywords:** interspecific hybrids, natural hybridization, *Saccharum asperum*, *Saccharum angustifolium*, *Saccharum villosum*, *Saccharum* × *officinarum*, geographic distribution, phylogeny

## Abstract

The commercial release of genetically modified organisms (GMO) requires a prior environmental and human/animal health risk assessment. In Brazil, the National Biotechnology Technical Commission (CTNBio) requires a survey of the area of natural occurrence of wild relatives of the GMO in the Brazilian ecosystems to evaluate the possibility of introgressive hybridization between sexually compatible species. Modern sugarcane cultivars, the focus of this study, derive from a series of hybridization and backcrossing events among *Saccharum* species. The so-called “*Saccharum* broad sense” group includes around 40 species from a few genera, including *Erianthus*, found in various tropical regions, particularly South-Eastern Asia. In Brazil, three native species, originally considered to belong to *Erianthus*, were reclassified as *S. angustifolium* (Nees) Trin., *S*. *asperum* (Nees) Steud., and *S. villosum* Steud., based on inflorescence morphology. Thus, we have investigated the potential occurrence of gene flow among the Brazilian *Saccharum* native species and commercial hybrids as a requisite for GMO commercial release. A comprehensive survey was carried out to map the occurrence of the three native *Saccharum* species in Brazil, concluding that they are sympatric with sugarcane cultivation only from around 14°S southwards, which precludes most Northeastern sugarcane-producing states from undergoing introgression. Based on phenology, we concluded that the Brazilian *Saccharum* species are unable to outcross naturally with commercial sugarcane since the overlap between the flowering periods of sugarcane and the native species is limited. A phylogenomic reconstruction based on the full plastid genome sequence showed that the three native *Saccharum* species are the taxa closest to sugarcane in Brazil, being closer than introduced *Erianthus* or *Miscanthus*. A 2-year study on eight nutritional composition traits of the 20 main sugarcane cultivars cultivated in Brazil was carried out in six environments. The minimum and maximum values obtained were, in percent: moisture (62.6–82.5); sucrose (9.65–21.76); crude fiber (8.06–21.03); FDN (7.20–20.68); FDA (4.55–16.90); lipids (0.06–1.59); ash (0.08–2.67); and crude protein (0.18–1.18). Besides a considerable amount of genetic variation and plastic responses, many instances of genotype-by-environment interaction were detected.

## Introduction

Genetically modified crops have become a useful tool in agriculture and are able to foster economic development, but they have stimulated public debate since their introduction in the 1990s (Mujjassim et al., 2019). Public acceptance is an important element for the success of a technology, and the consumers' opinion in relation to GMOs is based on ethical concerns and risk perception, because the licensed cultivars contain elements derived from genetically incompatible species, and may contain exogenous antibiotic or herbicide resistance genes of prokaryotic origins. Some of the concerns led 38 countries all over the world, including 19 in Europe, to prohibit officially the cultivation of GM crops, although they allow the import of both human food and animal feed derived from GM plants [International Service for the Acquisition of Agri-biotech Applications (ISAAA), 2017; Mujjassim et al., 2019].

Since the first release of a commercial GM crop, the “FlavrSavr®” tomato, in 1992, the adoption of this new technology has been quick. Until 2015, the main GM crops globally marketed were soybeans (cultivated in 95.9 million ha), maize (58.9 million ha), cotton (24.9 million ha), canola (10.1 million ha), and other minor crops, such as beets, alfalfa, papaya, pumpkin, eggplants, potatoes, apples, sugarcane, and poplar, which together correspond at most to 1.9 million ha [International Service for the Acquisition of Agri-biotech Applications (ISAAA), 2020]. All the main cultivated GM crops are propagated by seeds, facilitating the biosafety regulatory process, for once an event is licensed, the genotype can be introgressed into different focus varieties. For the vegetatively propagated crop species, there is a greater challenge, because the licensing is specific for each transgene insertion, that is, a new commercial licensing is necessary for each transformed cultivar, which becomes a limiting factor for the commercial releases.

In Brazil, sugarcane (*Saccharum* × *officinarum*) is a major crop, with 8.38 million ha planted (Companhia Nacional de Abastecimento, 2019), due to its great efficiency in biomass production and to its high sucrose content in the culms (Bonnett et al., 2008; Cheavegatti-Gianotto et al., 2011). However, the challenges with conventional breeding of this species should always be taken into account, mainly the complex genealogy, the polyploid and aneuploid nature of the highly yielding (in terms of biomass and sucrose content) commercial cultivars (Butterfield et al., 2001).

Due to the intrinsic difficulties of traditional sugarcane breeding, the development of cultivars by genetic modification, including gene editing, offers a great potential as it can overcome some of the limitations (Brinegar et al., 2017; Hilscher et al., 2017; Ricroch et al., 2017; Wang et al., 2017; Cristofoletti et al., 2018; Eriksson et al., 2018; Nerkar et al., 2018; Zhang et al., 2018; Khan et al., 2019). The commercial release of GM cultivars is conditioned to the assessment of biosafety risks (Cheavegatti-Gianotto et al., 2011), to meet the requirements of the national regulatory systems (Jaffe, 2004; Eriksson et al., 2018; Khan et al., 2019).

In Brazil, the National Biotechnology Technical Commission (CTNBio) has approved more than 21 contained field trial releases of GM sugarcane in the environment in the last 2 years. These GM approvals are being tested in the field for insect resistance, glyphosate tolerance, biomass yield increase, and tolerance to abiotic stresses such as water deficit (information obtained from the company AgroBio Brasil). The first Brazilian GM sugarcane cultivars (“CTC 20 Bt,” “CTC 9001 Bt,” and “CTC 93309-4 Bt,” from the Center of Sugarcane Technology [CTC], Piracicaba, SP, Brazil), which are resistant to the sugarcane borer (*Diatraea saccharalis*) have already been approved and released for commercial cultivation (Cheavegatti-Gianotto et al., 2018). A rigorous, multidisciplinary risk assessment process, aiming at the potential impact on the environment and at food safety must be followed before the commercial release of GM cultivars and their progeny occur. The risk analyses related to both environmental and food safety required in Brazil addresses the potential of involuntary gene transfer to related species, which might cause negative effects (Ellstrand, 2003; Anderson and Vicente, 2010; Jong and Rong, 2013). We assumed a logical chain of requirements of different natures that ought to be attended should gene flow take place: (1) Species evolutionarily close to the crop are identified (*the phylogenetic requirement*); these species are the main candidates to involvement in gene flow; (2) The occurrence of the candidate wild species is mapped (*the geographical requirement*). The wild and crop species should be sympatric; (3) The wild and the crop species should flower synchronously (*the temporal requirement*); (4) The wild and the crop species should be reproductively compatible (*the physiological/genetic requirement*), which means that they have to be both sexual, produce viable pollen and the pollen tubes of one species have to be able to deliver the male gametes to the embryo sac of the other, producing a viable embryo; (5) Interspecific reproduction should occur spontaneously in the habitat of the species involved (*the ecological requirement*), which means that pollen is successfully transferred and the species are syntopic. In addition to gene flow studies, analyses also assess the existence of substantial equivalence between the GMO and its parental organism, in the case of GM species used as food and/or feed, to guarantee that no trait other than the target has been introduced inadvertently.

In the case of genus *Saccharum*, native species (*S. angustifolium, S. asperum*, and *S. villosum*) occur in several Brazilian regions and are reported in floristic surveys (Filgueiras and Lerina, 2001; Carporal and Eggers, 2005; Kameyama, 2006). In spite of the economic importance of some of the species of the genus, there still are controversies about their taxonomic circumscription and the overall organization of the taxon (Welker and Longhi-Wagner, 2012). The genus *Saccharum lato sensu* includes species of *Erianthus* Michx., and encompasses *ca*. 40 species (Clayton and Renvoize, 1986). However, some authors consider *Erianthus* as distinct from *Saccharum* (Watson et al., 1992; Soreng et al., 2015) and *Tripidium* (Lloyd Evans et al., 2019; Welker et al., 2019) and *Lasiorachis* (Vorontsova et al., 2019) as separate genera. The phylogenetic analysis performed by Hodkinson et al. (2002) did not find any justification for this division, but these authors did not include all species of *Erianthus* in the study. Here we will adopt the circumscription of *Saccharum* in its wider sense, following Filgueiras and Lerina (2003). In Brazil there are three native species of *Saccharum*, previously classified as *Erianthus*: *S*. *angustifolium* (Nees) Trin., *S. asperum* (Nees) Steud., and *S. villosum* Steud. (Filgueiras and Welker, 2013). The information about these species, however, is scarce and is normally present only in floristic surveys (Cheavegatti-Gianotto et al., 2011). Grasses are commonly identified on the basis of their floral characters, which may constitute a problem, since the inflorescence does not persist for a long period of the life cycle of the plants. In the genus *Saccharum*, leaf blade morphology and pilosity are also of taxonomic importance (Welker and Longhi-Wagner, 2012), but during some periods the leaves become dry and do not keep their characteristics.

Extensive botanical information about these species is lacking (Cheavegatti-Gianotto et al., 2011). The genetic studies on the genus *Saccharum* (*lato sensu*) are complex because of the evidence of multiple cycles of past polyploidization events and consequent reticulate evolution, often followed by silencing and elimination of duplicated genes. Thus, phylogenetic reconstruction involving the genera close to *Saccharum* or even the *Saccharum* species, and especially the cultivated hybrids (*Saccharum* × *officinarum*) are challenging, especially if nuclear DNA sequences are used (Garsmeur et al., 2018; Zhang et al., 2018; Souza et al., 2019). Phylogenomics based on chloroplast genome (plastome) sequences may be a solution to overcome the difficulties imposed by polyploidy and aneuploidy, both found in the genus *Saccharum* (*lato sensu*), because the plastome is not affected by the ploidy level.

This work had two main objectives. First, to evaluate the potential for gene flow between three Brazilian wild species of *Saccharum*, and Brazilian commercial sugarcane cultivars, based on genetic relatedness estimated by genome-level phylogenies and by the detection of sympatry. The second objective was to establish a nutritional compositional information database of the principal Brazilian commercial sugarcane cultivars grown in different environments (regions and years), which can be compared with other databases. More detailed studies carried out on the part of the authors, related to the degree of overlap among flowering times, to pollen fertility, and sex distribution (of sugarcane), to the geographic distribution and the prediction of ecological niches of the wild and domesticated species are being or will soon be submitted elsewhere and will expand the scope of this study.

## Materials and Methods

### *Saccharum* Species and Cultivars

The choice of the Brazilian wild species of *Saccharum* for the geographic distribution study was based on their a priori potential for crossing with sugarcane, which is related to their evolutionary closeness to the crop. There are three species indicated by agrostologists as close relatives of sugarcane: *S*. *angustifolium* (Nees) Trin., *S. asperum* (Nees) Steud., and *S. villosum* Steud. (Filgueiras and Welker, 2013).

For the phylogenetic analysis by Ultra-Barcoding, based on the chloroplast full genome sequence (Kane et al., 2012), total DNA from leaves of the following materials were utilized: the commercial cultivar SP80-3280; two parental species (*S. officinarum*—Muntok Java; *S. spontaneum*—SES 208), collected in the germplasm bank of the Plant Breeding Laboratory, Center of Nuclear Energy in Agriculture (CENA/USP), Piracicaba, SP, Brazil; three Brazilian wild species (*S. angustifolium*; *S. asperum*, and *S. villosum*), collected in the metropolitan region of São Paulo. In addition, *S. bengalensis* (“US4714”) and *Miscanthus nepalensis* (“IND82318”), collected from the germplasm bank of the Center of Sugarcane Technology (CTC), Camamu, BA, Brazil, both from the *Saccharum* Complex. For the following accessions, chloroplast genome sequences are available at GenBank (NCBI): cultivars *S*. × *officinarum* “SP80-3280” (accession AE009947.2) and *S*. × *officinarum* “NCo310” (AP006714.1); *S. arundinaceum* “JW630” (LC160130.1), *Miscanthus sacchariflorus* (NC_028720.1), *Miscanthus sinensis* “Niigata 410” (LC160131.1), and *Sorghum bicolor* “BTx623” (CM000760.3).

For the nutritional composition trials, a collection of 20 commercial sugarcane varieties from Syngenta Cultivar Protection, Itápolis, SP, Brazil, and from CTC were used. The criteria for the choice of the cultivars evaluated were the proportion of planted area in Brazil (relevance), maturation time and adaptability to different production environments. The trials were divided according to the maturation period of the cultivars, *viz*., early (eight cultivars: RB855156, RB855453, RB965917, RB966928, CV7231, CTC9, CTC17, and CTC21) and middle/late (12 cultivars: RB92579, RB835054, RB867515, RB965902, IACSP955000, IACSP955094, CV7870, SP81-3250, SP83-2847, CTC4, CTC15, and CTC20), encompassing, thus, genotypes of the main Brazilian sugarcane breeding programs.

### Mapping the Species Occurrence

**Figure 1:**
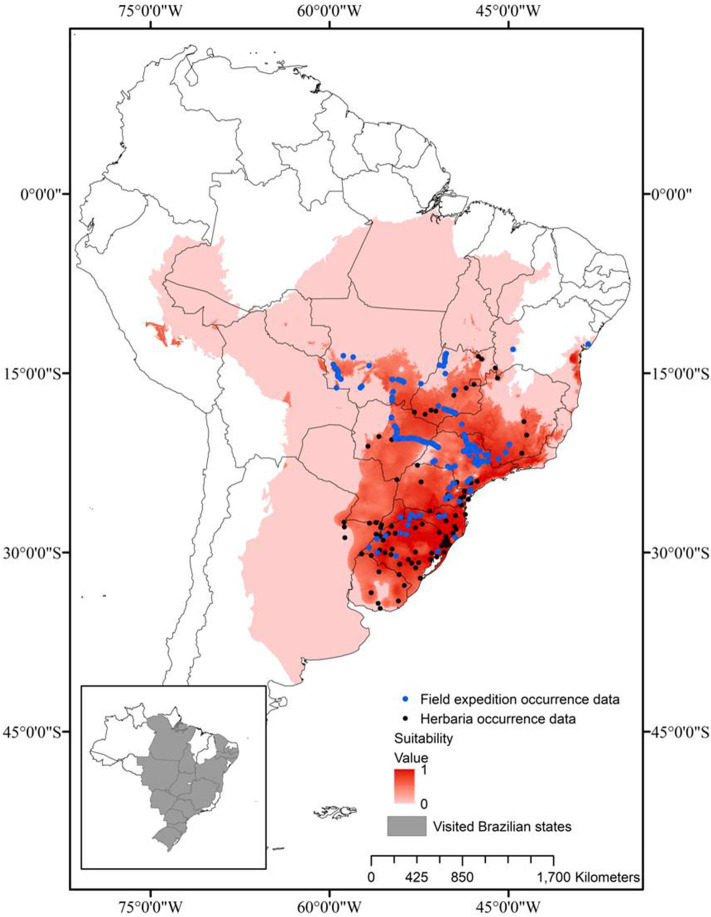
Prediction of the ecological niche of the Brazilian native species of *Saccharum*, based on geographic distribution data (black dots) from previous collecting travels (states visited in gray, in the inset), herbarium data and the literature.

In order to delimit the occurrence of the three Brazilian species of *Saccharum*, a geographic data gathering was conducted both in digitalized/online and non-digitalized international and local herbaria, complemented by field mapping/collecting expeditions in many states of Brazil. Utilizing the main roads of the country, all the regions (South, Southeast, Center-West, North, and Northeast) were visited and a total of 115 sites, with populations of the three species, were mapped (Figure 1) and their geographic coordinates were registered with a Garmin GPS 76 (Jaryan et al., 2013).

### Distribution Modeling

A niche-prediction model was proposed, based on the raw occurrence data, that helped both describe the biogeography of the species and provide guidance for further collecting efforts, in a iterative way. The Maxent 3.4.1 software (Phillips et al., 2006) was used to generate the distribution models of *Saccharum* species native to Brazil. The GPS coordinates of the 115 points obtained in the collecting expeditions were used as input, together with the points of occurrence obtained from GBIF (Global Biodiversity Information Facility; www.gbif.org). In addition to species occurrence data, environmental data were also used as input for the construction of the distribution model. Data for 19 bioclimatic parameters were downloaded from the Worldclim version 2.0 data portal (www.worldclim.org) for the study area (Table 1). These data were downloaded and used in the model (Hijmans et al., 2005), after being converted from “GRID” to “ASCII” format by Arc GIS v. 10.6 (ESRI, Redlands, CA, EUA; Scheldeman and Zonneveld, 2010) in order to generate data compatible with MaxEnt. A Pearson correlation test, using the R software (3.4.1; R Core Development Team, 2017), was performed among the 19 bioclimatic variables, with only those variables with correlation coefficients ≤0.9 being used for the generation of models, since the autocorrelations between the predictive variables were verified as a recognized source of error (Dormann et al., 2007). Thus, six bioclimatic variables were used to generate the final models (annual mean temperature; mean diurnal range; annual precipitation; precipitation seasonality; precipitation of warmest quarter and precipitation of coldest quarter).

**Table 1:** The 19 bioclimatic factors tested as model inputs.

**Code**	**Parameter**
Bio 1^*^	Annual mean temperature
Bio 2^*^	Mean diurnal range
Bio 3	Isothermality
Bio 4	Temperature seasonality
Bio 5	Maximum temperature of warmest month
Bio 6	Minimum temperature of coldest month
Bio 7	Temperature annual range
Bio 8	Mean temperature of wettest quarter
Bio 9	Mean temperature of driest quarter
Bio 10	Mean temperature of warmest quarter
Bio 11	Mean temperature of coldest quarter
Bio 12^*^	Annual precipitation
Bio 13	Precipitation of wettest month
Bio 14	Precipitation of driest month
Bio 15^*^	Precipitation seasonality
Bio 16	Precipitation of wettest quarter
Bio 17	Precipitation of driest quarter
Bio 18^*^	Precipitation of warmest quarter
Bio 19^*^	Precipitation of coldest quarter

* *Indicate variables used as model inputs*.

The parameters utilized in the construction of the Species Distribution Models were: convergence threshold of 1e^−5^, 500 iterations and 10,000 background points. Each model was subjected to ten repetitions, validated by the bootstrap method. The presence points selected for the generation of the model (70% of total) were partitioned again into two groups, 70% of the occurrence points having been used for training the model, and the remaining 30%, for its internal test. The models were evaluated with the AUC (Area Under the Curve) index. The omission values and the *p*-value were utilized for three cutting thresholds: the 10-percentile training presence Clog-log threshold, the Maximum test sensitivity plus specificity Clog-log threshold and the Minimum training presence Clog-log threshold. The threshold with the least omission values was chosen for the final model. The contribution of the six variables to the final model was tested with the jackknife method. Response curves were generated for the two variables that contributed most to the model.

### Phylogenetic Analysis: DNA Extraction

Total DNA was extracted from fresh leaf tissue, with the DNeasy Plant Mini Kit (Qiagen, Valencia, California, EUA). The quality of genomic DNA was evaluated by electrophoresis in 0.8% agarose gel stained with SYBR gold (Invitrogen, Molecular Probes, Eugene, Oregon, USA). DNA concentration was determined by fluorometry (DyNA Quant 2000 Fluorometer, Amersham Biosciences, Buckinghamshire, UK) and with a NanoDrop spectrophotometer (Thermo Scientific, Wilmington, USA).

### Phylogenetic Analysis: Chloroplast DNA Sequencing

DNA samples were fragmented by sonication (400- to 500-bp), and the fragments were ligated with adaptors using the Nextera DNA sample preparation kit (Illumina). The chloroplast genomes of *S. angustifolium, S. asperum*, and *S. villosum*, the cultivar SP80-3280, the parental species *S. officinarum* (cv. Muntok Java) and *S. spontaneum* (accession “SES208”), as well as *Miscanthus nepalensis* (“IND82318”), and *S. bengalensis* (“US4714”) were sequenced with an Illumina HISEQ2500 platform (Atherton et al., 2010; Nah et al., 2015; Daniell et al., 2016; Dierckxsens et al., 2017), with DNA Single Read or Paired End, Module HIGH OUTPUT—Paired End 2 × 100 pb, and a 100-million-read cover per library, in the Central Laboratory of High-Performance Technologies in Life Sciences (LaCTAD) of State University of Campinas (UNICAMP), Campinas, SP, Brazil. The sequences of the other species were obtained from the Internet databases. With these sequences, it was possible to assemble the plastid genome of part of those species that compose the genus *Saccharum*, including the three Brazilian *Saccharum* species in the phylogenomic analysis. The *Saccharum* plastid genome was assembled based on the published sequence of the “NCo310” (GenBank AP006714.1) sugarcane hybrid (Asano et al., 2004). At the end, the total cover was 14 times as long as the chloroplast genome length.

### Phylogenetic Analysis: Phylogeny Reconstruction

The amino acid sequences codified by all the genes present in 13 chloroplast genomes were concatenated and then aligned according to the standard configuration of the Muscle Alignment tool in Geneious R9.1 (Kearse et al., 2012). The amino acid substitution model Blosum62+I+G+F was indicated as the most adequate by the software ProtTest (Abascal et al., 2005) and a maximum likelihood phylogenetic tree was generated with 1,000 bootstrap repeats by RAxML v. 7.7.8 (Stamatakis, 2006). The analyses involving the structural similarities among chloroplast genomes of the “*Saccharum* broad sense” and their phylogenetic relationships utilized the sorghum cultivar BTx623 as an outgroup.

### Nutritional Composition: Field Experiment

The cultivar trials were performed in six environments, which represent the main sugarcane cultivation regions in Brazil: Conchal [22°24′S; 47°06′W; 591 m above sea level (asl), State of São Paulo], Jaboticabal (21°16′S; 48°23′W; 615 m asl, State of São Paulo), Taciba (22° 23′S; 51°17′W; 416 m asl, State of São Paulo), Rolândia (23°18′S; 51°22′W; 730 m asl, State of Paraná), Montividiu (17°26′S; 51°10′W; 821 m asl, State of Goiás), and Carpina (07°35′S; 34°15′W; 184 m asl, State of Pernambuco). The climate is classified as Aw in Jaboticabal, Montividiu and Carpina, Cfa in Taciba and Rolândia and Cwa in Conchal, according to the Köppen scale (Köppen, 1936; Kottek et al., 2006). In each environment, the experiment was performed in a randomized blocks design, with three replications. The experimental plots consisted of two parallel ranks 3 m long and 1.4 m apart. The weed, fertilizer, and pest management were done according to local commercial agricultural practice. The experiments were set up in 2014 and there were two annually harvested crops: first-year crop (2015) and first-ratoon crop (2016). Each sample collected for the nutritional and technological composition assessments was made of 10 entire culms, including their culm tips.

### Nutritional Composition: Chemical Analyses

The analyses were performed at the Technological Analyses and Simulation Laboratory (LAST) of the Agricultural Sciences Center, Federal University of São Carlos, in Araras, State of São Paulo, Brazil. Sample composition was analyzed according to the recommendations of the Organization for Economic Co-operation and Development (OECD) (2011) for sugarcane, which has sugars as its main derived product. However, the OECD recommends that some sugarcane constituents be measured in entire culms, including the leaves. The culms had their composition analyzed in terms of: moisture [AOAC (Association of Official Analytical Chemists, https://www.aoac.org) 935.29], crude protein (AOAC 2001.11), fat ether extract (lipids) (AOAC 2003.06), crude fiber (Fiber % Cana Tanimoto, Tanimoto method, ABNT NBR16225), fiber in neutral detergent—FND (Ankom method 13), fiber in acid detergent—FAD (Ankom method 12), ash (AOAC 942.05), and sucrose (Pol % Cana Tanimoto, Tanimoto method ICUMSA, method GS5/7-28, 2013). To summarize the sugarcane nutritional composition essays, descriptive statistics, and graphical procedures were performed. For each trait, minimum, maximum, average, confidence interval for average at 95%, and standard deviation of the mean were calculated. Also, limits defined by three times the standard deviation from the mean were calculated to infer the range that encompasses 99% of the data. In order to better understand the data distribution, skewness, and kurtosis were calculated using the package *agricolae* (version 1.3.1) and the graphical representation was done using the package *ggplot2* (version 3.2.1), both run on *R package* (3.6.1; R Core Development Team, 2017).

## Results and Discussion

### Occurrence of the Brazilian Species and Niche Prediction

The Brazilian species of *Saccharum* have a regional distribution: *S*. *angustifolium* (Nees) Trin. occurs in the Southeast and South regions of Brazil, *S. asperum* (Nees) Steud. occurs from the Center-West to the South and *S. villosum* Steud., the most widely distributed, is present from the Northeast to the South (Filgueiras and Welker, 2013). The distribution model that predicts the habitat and the niche of a species depends on the refinement of the variables and the validation tests, but these frequently present distortions (Phillips et al., 2006; Kamyo and Asanok, 2019).

In this study, the six variables most adequate for the determination of the distribution model of the three Brazilian native *Saccharum* species were: (a) annual mean temperature (BIO01); (b) mean diurnal range (BIO02); (c) annual precipitation (BIO12); (d) precipitation seasonality (BIO15); (e) precipitation of warmest quarter (BIO18); (f) precipitation of coldest quarter (BIO19). The climatic patterns establish the distribution limits of the plant taxa at a regional-global level (Shimwell et al., 1982; Woodward, 1987; Prentice, 1992; Taylor and Hamilton, 1994). The most important variables for the construction of the distribution model of the Brazilian native *Saccharum* species were the average annual temperature and the annual rainfall, which together explained 74.3% of the species distribution. These results indicate that rainfall has a crucial role in the distribution of these species, especially because they grow mainly in wetlands of warm regions. Similar results were found, for instance, for the distribution model of *Dipterocarpus alatus* in central Thailand (Kamyo and Asanok, 2019).

### The “Area Under Curve” (AUC) Analysis

The model for the Brazilian *Saccharum* species had an AUC of 0.8586 (±0.019). The cutting threshold chosen was the 10th-percentile training presence threshold, since this threshold gave the best results when the balance between omission and overprediction errors was considered. An AUC value of 0.50 indicates that the model should be considered random and a bad predictor, while a value of 1.00 represents excellent precision (Swets, 1988).

The results of the distribution model must be rigorously assessed, because the ecological niche of a species covers an area wider than the geographic zone the species occupies and not all the suitable areas are inhabited (Kamyo and Asanok, 2019). The populations collected in great part of the country, mentioned in Materials and Methods, were utilized in the validation of the distribution model.

Based on the information collected, the suitability threshold of the distribution model was 0.31 and the omission percentage was 9.47%. The distribution model generated by MaxEnt 3.4.1 was highly satisfactory, indicating that 40.1% of the sampling points are located within an area of high suitability (*x* > 0.75), 43.5% have 0.75> *x* ≥0.50 and only 16.4% are located in unsuitable areas, with 0.5> *x* ≥0.31. As a contrasting example, Kamyo and Asanok (2019) report for *D. alatus* that, for an area of 53,483 km^2^, only 5.84% (704.27 km^2^) were highly suitable, 14.59% (1,757.37 km^2^) was suitable, 24.83% (2,991.10 km^2^) was moderately suitable and 54.72% (6,592.02 km^2^) was unsuitable for the species *D. alatus*.

On the basis of both the distribution model of the three native species and the mapping expeditions, it was evident that the wild populations are sympatric in relation to sugarcane only south of the parallel 14°S, which excludes most of the sugarcane cultivation area in the Northeast, significantly reducing the possibility of introgression.

During the mapping travels throughout Brazil, other kinds of information were gathered, such as the existence of three categories of population, according to size, and stability. First, species may form large, stable populations in humid environments, near brooks, and rivers. The second type refers to small populations, with just a few dozens of individuals, and occupies suboptimal or relatively unfavorable environments, generally disturbed by humans and unstable, where the original vegetation has been partly or totally removed. Thus, the three species may all be classified as invasive, and display putative adaptations for this condition, such as the trichomes on the spikelets and the reproductive system. The third type of population is composed sometimes by one or two individuals, or a few more, and frequently they are very isolated from the larger populations, sometimes settling in suburban zones. They can grow even on ravines or other disturbed terrain, generally in the crevices that can retain rain water for longer.

There is, thus, a high probability that the population dynamics of these wild species fit the classic source-sink model (Pulliam, 1988; for a recent application, see Seipel et al., 2016), where seeds of the central, stable populations, the “sources,” disperse over long distances and found a great many unstable populations, the “sinks,” that receive migrants regularly, although they have high mortality and are unable to conserve their numbers by themselves. The possibility cannot be discarded that the secondary populations go extinct frequently and are constantly refounded.

The Brazilian wild species of *Saccharum* do not reproduce by cross-pollination (manuscript in preparation), although it is not yet known whether they are autogamous or agamospermic. Both hypotheses will be tested by progeny analysis in a subsequent study. The seeds of the Brazilian *Saccharum* species are formed very early, when the inflorescence is still deep inside the rolled flag leaf; the flowers are very small. Curiously, the seeds are not dormant, an atypical characteristic for an invasive plant, for which dormancy (dispersal through time) is very advantageous (Leverett and Shaw, 2019). Because the Brazilian wild *Saccharum* plants do not have vegetative propagation mechanisms, such as stolons or rhizomes, they depend exclusively on seeds for colonizing new areas.

### Phylogenomics

Many wild species of *Saccharum* relatives, including the Brazilian wild species, are allopolyploid (Welker et al., 2015). The three Brazilian *Saccharum* wild species are distinct species; however, there is evidence that natural hybrids between *S. angustifolium* and *S. villosum* may occur (Filgueiras and Welker, 2013), which might be explained by local chasmogamous mutants, phenotypic plasticity or even natural intraspecific variation. Phylogenomics based on whole plastomes allowed us to show the relationships between species and in the future, as we add infraspecific taxa, it may allow us to include individual populations, interspecific hybrids and geographic races as well, in order to assist in the characterization and conservation of the three species.

The *Saccharum* plastid genome sizes ranged from 141,182 bp (*S. asperum*) to 141,869 bp (*S. bengalensis*—US4714), and all the genomes presented typical circular structures, with two-inverted repeat sequences (all the chloroplast genomes sequenced are in Supplementary Materials 1–8). The number of Single Nucleotide Polymorphisms in relation to the plastid reference genome of “NCo310” ranged from three (*S. bengalensis*—US4714) to 355 bp (*Miscanthus nepalensis*—IND82318). The number of SNPs was 96 for *S. angustifolium*, 197 for *S. villosum*, and 207 for *S. asperum*.

Gene number in the plastid genome was the least variable component (199 in *S. villosum*, to 204 in *S. asperum*; *S. angustifolium* has 201). GC content also varied little, from 38.3% in *S. angustifolium* to 38.5% in *S. bengalensis*—US4714). These values were similar to those of other Panicoideae, including *S. officinarum* (Asano et al., 2004), *Miscanthus sinensis* (Nah et al., 2015), *Sorghum bicolor* (Saski et al., 2007), *Erianthus arundinaceus* and *Miscanthus sinensis* (Tsuruta et al., 2017).

### Comparison of the Chloroplast Genome of “*Saccharum* Broad sense” and *Sorghum*

**Figure 2:**
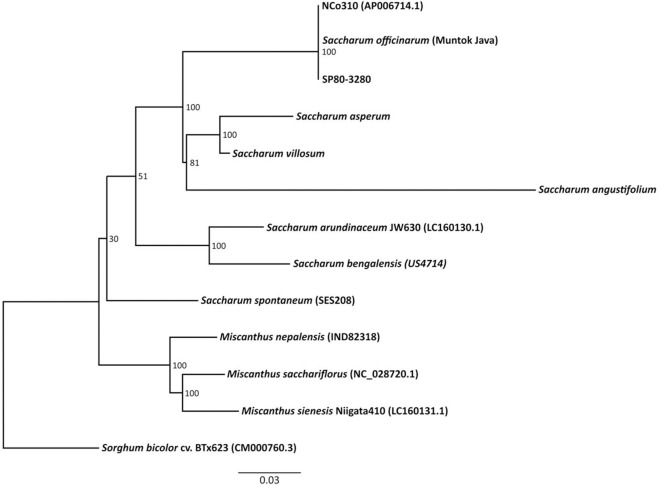
Phylogenetic analysis of 11 species and two sugarcane hybrids, including three *Miscanthus* species that are included in the *Saccharum* complex of wild relatives and *Sorghum bicolor* as an outgroup.

The chloroplast genomes of the “*Saccharum* broad sense” and of the outgroup *Sorghum bicolor* cv “BTx623” (GenBank #CM000760.3) were aligned (Figure 2). The Maximum Likelihood (ML) analysis resulted in a single tree. From the nine nodes, six have bootstrap support values of 100% (Figure 2). The Maximum Parsimony analysis generated one single tree, and both the ML and the MP trees have a similar topology, mostly congruent with the published grass trees based on complete chloroplast genomes (Young et al., 2011; Wu and Ge, 2012; Gao et al., 2014; Lózsa et al., 2015; Tsuruta et al., 2017).

The Brazilian wild *Saccharum* species appeared as closely related to the other *Saccharum* species, and are the taxa genetically closest to *S. officinarum, S*. × *officinarum*, and *Miscanthus*. This result disagrees with the scheme proposed for the evolutionary history of the hybrids (Ferrari, 2010). Our results also differ from those of Sobral et al. (1994) who, based on a study on 32 genotypes of the *Saccharum* complex using phylogenetic analysis of chloroplast restriction enzyme site mutations, showed that *Erianthus* diverged from other lineages early in the evolution of subtribe Saccharinae. The result also differs from those found by Tsuruta et al. (2017) that showed that the *S. bicolor* chloroplast genome is more closely related to that of *Saccharum* than to that of *Erianthus*. Discrepancies in phylogenies are expected whenever different materials and methods are used. In the cases above, different subsets of Saccharinae species were compared and different techniques were used to generate characters (chromosome morphology, restriction sites, whole chloroplast genome). However, it is noteworthy that in our study, the three Brazilian native species of *Saccharum* were compared to other species close to sugarcane and were found to be the closest, excepting naturally one of the ancestors of the crop.

Our study supports that the Brazilian wild species of *Saccharum* are the Brazilian Saccharinae most closely related to sugarcane, which supports our decision to include these species in this study.

### Nutritional Composition

The concept of substantial equivalence was recognized by Organization for Economic Co-operation and Development (OECD) (1993) to ensure that new foods derived from biotechnology be as safe as their conventional counterpart [Organization for Economic Co-operation and Development (OECD), 2011]. This concept was then enhanced through the Codex Alimentarius Commission [founded by the Food and Agriculture Organization, of the United Nations (FAO) and the World Health Organization (WHO)], that developed food standards, guidelines, codes of practice, and other relevant documents under the FAO-WHO Food Standards Programme. In the specific case of sugarcane, the OECD recommends that a new cultivar be analyzed in relation to its contents of main components (humidity, raw protein, lipids, ash, fibers, and sucrose) [Organization for Economic Co-operation and Development (OECD), 2011]. There is local literature on the topic (Azevêdo et al., 2003; Santos and V; Evangelista, 2006; Anjos et al., 2007), but most of the studies are about the use of sugarcane as silage and, more recently, about the release of transgenic cultivars (Gianotto et al., 2019). Nowadays there is no base-line information on nutritional composition of Brazilian sugarcane cultivars, as is required and recommended by the OECD. When we compare the results of the present study with the values previously published by Organization for Economic Co-operation and Development (OECD) (2011), some differences in the minimum and maximum values were identified in, for instance, traits associated with fiber, such as crude fiber (8.06–21.03, our study) vs. (22.7–35.9, OECD), FDN (7.20–20.68, our study) vs. (39.4–77.6, OECD), FDA (4.55–16.90, our study) vs. (24.3–54.4, OECD). Differences in value ranges were also observed for lipids (0.06–1.59, our study) vs. (0.8–1.3, OECD), ash (0.08–2.67, our study) vs. (1.2–6.2, OECD), and crude protein (0.18–1.18, our study) vs. (1.8–4.1, OECD) (the nutritional composition data can be found in Supplementary Material 9). These variations may be due to different environmental conditions, genetic background and interference of genotype × environment (G × E). These results highlight the importance of developing databases of percent nutritional composition obtained with cultivation conditions found in Brazil so that the phenotypic ranges observed can serve as comparative values when GM cultivars are assessed there. This reinforces that substantial equivalence assessments should be performed considering databases obtained from sites as close as possible to those where the GMO is to be used.

One of the priority points in substantial equivalence studies is the possible interference of genotype × environment (G × E), which is frequently an important source of variation in sugarcane cultivars observed in many breeding programs all over the world and constitutes a complicating factor during the selection of clones (e.g., Kang and Miller, 1984; Milligan et al., 1990; Jackson and Hogarth, 1992; Ramburan et al., 2011; Chen et al., 2012). The differential behavior of genotypes in different environments, i.e., the genotype-by-environment interaction, results in alterations in the genotype ranking in competition trials or a change in values of the differences between genotypes in different localities. In general terms, the G × E interaction corresponds to the differential response of the genotypes to changes in the environments, thus evidencing the dependence between genetic and environmental effects. The importance of the study on G × E interactions is well-recognized (Kumar et al., 2018). In this context, the cultivars exposed to some kind of stress may show a wide range of complex and variable responses which depend on the genotype's inherent sensitivity to stress (Chen et al., 2012).

The information on nutritional composition and other components present in both fresh and processed sugarcane is necessary for the development of programs in many areas, such as nutrition, agriculture, industry and food commerce (Giuntini et al., 2006), as well as for being utilized as reference in biosafety assessments of GM cultivars. Although there are many articles about G × E interactions influencing production variables (tons of sugarcane per hectare, Pol per hectare, etc.), there is no report about G × E interaction assessments in variables associated to the nutritional composition in sugarcane.

**Figure 3:**
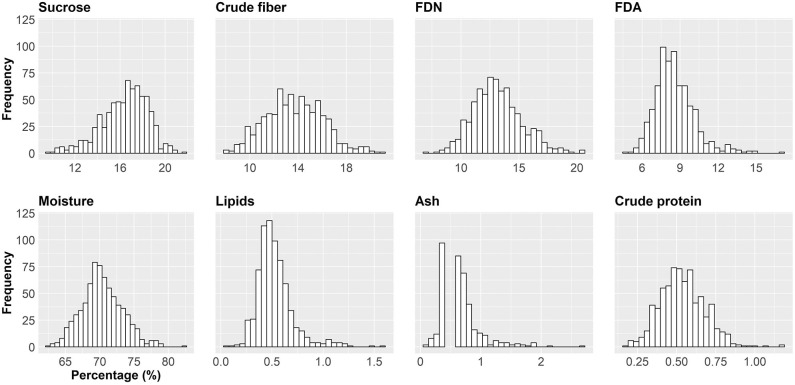
Histogram for the eight sugarcane nutritional composition traits (NC). Axis *x* shows the intervals of the trait indicated in percentage. Axis *y* indicates the absolute frequency (the number of the smallest experimental units) per interval. The smallest experimental unit is each of the replicates of a cultivar/region/year combination.

**Figure 4:**
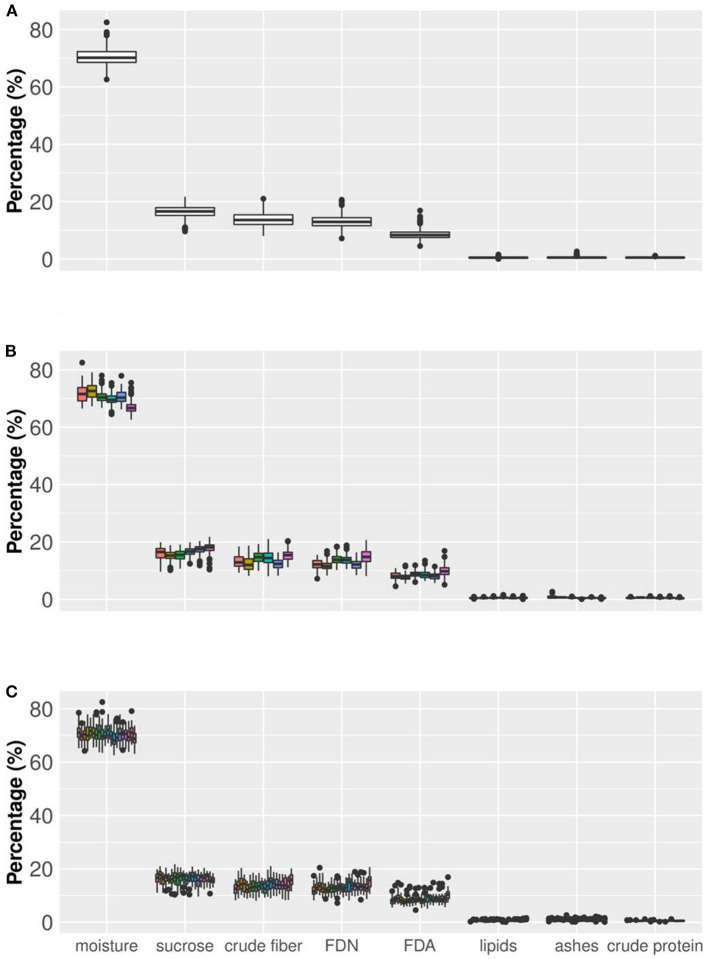
Boxplot of sugarcane nutritional composition traits (axis *x*). Axis *y* indicates the values of the traits, all expressed in percentage. Box edges represent the upper and lower quartile with median value shown as bold line in the middle of the box. **(A)** Boxplot considering all the information for eight nutritional components (NC) (20 varieties and six production environments in two crop years and three replicates). **(B)** Boxplot showing the behavior of NC traits considering six environments (from left to right, Jaboticabal, Conchal, Rolândia, Taciba, Montividiu, and Carpina). **(C)** Boxplot considering the phenotypic variation of the 20 varieties across all production environments (from left to right: CTC04, CTC09, CTC15, CTC17, CTC20, CTC21, CV7231, CV7870, IACSP955000, IACSP955094, RB835054, RB855156, RB855453, RB867515, RB92579, RB965902, RB965917, RB966928, SP813250, and SP832847).

In this paper, we show an important effort to build profiles of nutritional composition in Brazilian sugarcane cultivars. For this, we set up an experiment across six representative production environments in Brazil, with two harvests (one harvest per year) and varieties from different genetic backgrounds. We evaluated 720 datapoints (20 varieties evaluated at six locations and 2 years with three replicates) that were summarized in Table 2 and Figures 3, 4.

**Table 2:** Descriptive statistics for sugarcane nutritional composition traits, as well as skewness and kurtosis estimates.

**Traits**	**Min**	**Max**	**Average**	Confidence interval^**^		$\bar{x}$ ± 3 × sd ^***^		**Skewness**	**Kurtosis**
			(SEM^*^)	**Lower**	**Upper**	**Lower**	**Upper**		
Moisture	62.60	82.50	70.37 (0.11)	70.08	70.66	61.47	79.27	0.31	0.24
Sucrose	9.65	21.76	16.39 (0.07)	16.19	16.59	10.25	22.53	−0.57	0.27
Crude fiber	8.06	21.03	13.72 (0.08)	13.49	13.95	6.62	20.82	0.16	−0.25
FDN	7.20	20.68	13.15 (0.07)	12.95	13.35	6.81	19.49	0.45	0.32
FDA	4.55	16.90	8.58 (0.05)	8.43	8.73	3.90	13.26	1.08	2.51
Lipids	0.06	1.59	0.53 (0.0)	0.51	0.55	0.00	1.06	1.62	4.87
Ash	0.08	2.67	0.59 (0.01)	0.56	0.62	0.00	1.39	2.70	11.39
Crude protein	0.18	1.18	0.54 (0.0)	0.52	0.55	0.11	0.97	0.45	0.80

* *SEM: standard error of the mean. Rounded to two decimals*.

** *Confidence Interval obtained at 99%*.

*** *Three times the standard deviation from the mean (x), may contain 99% of data*.

The raw data for each trait was analyzed using descriptive statistics, such as minimum, maximum, average ($\bar{x}$) and mean confidence interval (Table 2). Limits were defined by three times the standard deviation from the mean ($\bar{x}$ ± 3 × sd). Values of skewness and kurtosis were also estimated. Essentially, two major kinds of information were inferred for the traits, *viz*., the average and the confidence interval. For example, if one considers moisture, the average was 70.37% with standard error of the mean of 0.11. The confidence interval at 99% varied from 70.08 to 70.66%. Still, the minimum observed was 62.20% and the maximum was 82.50%. To suggest outlier candidates, lower, and upper limits defined by a range of ($\bar{x}$ ± 3 × sd) can be used. In this case, values varied from 61.47 to 79.27%, suggesting that the maximum value could be an outlier. The same idea can be applied for others traits, i.e., sucrose content showed average of 16.39 and ($\bar{x}$ ± 3 × sd) limits of 10.25 and 22.53; for crude fiber, FDN, FDA, lipids, ash, and crude protein, average values of 13.72 (6.62 and 20.82), 13.15 (6.81 and 19.49), 8.58 (3.90 and 13.26), 0.53 (0.00 and 1.06), 0.59 (0.00 and 1.39), 0.54 (0.11 and 0.97) were estimated, respectively. In general, extreme values (outliers) out of the range ($\bar{x}$ ± 3 × sd) have been observed for all traits.

The histograms in Figure 4 provide a visual aid for overviewing the dataset distribution. Briefly, the data pattern suggested a normal distribution for the eight NC traits. Lipids was the trait with maximum concentration of data around the average. In contrast, crude protein had the wider distribution with the shortest peak for the mode. Asymmetry is also suggested for all variables. The distribution shape and asymmetry were quantified by skewness and kurtosis estimates (Table 2). For skewness, values close to zero indicate symmetrical distribution. Here, the trait with the most symmetrical distribution was crude fiber (0.16), followed by moisture (0.31), FDN (0.45), and crude protein (0.45). Ash (2.70), lipids (1.62), and FDA (1.08) were the most positively skewed, i.e., with the majority of the data concentrated on the left. The only trait with negative skewness was sucrose (−0.57). It should be stressed that, along the history of sugarcane breeding, breeding programs have focused on selecting genotypes with increasing ability to accumulate sucrose (Morais et al., 2015; Balsalobre et al., 2016; Kumar et al., 2018). With this in mind and considering that the 20 varieties selected had the ability to yield high sucrose content, a left skewed distribution was expected. On the other hand, moisture and fiber-derived traits were not major focuses for selection, which reflected in traits less skewed. The higher absolute values of skewness were obtained for ash and lipids, whose contributions to NC were very small. Kurtosis estimates also provide insights about data variability; e.g., the highest values were found for ash (11.39), lipids (4.87), and FDA (2.51) indicating a concentration toward the mean. On the other hand, the lowest value of kurtosis was found for crude fiber (−0.25) whose distribution was wider than those of the other traits. Moisture (0.24) and sucrose (0.27) showed intermediate values. Considering that our dataset represents the interaction of both Brazilian genetic background and the environmental conditions for sugarcane-producing areas for two crop years, it is possible to infer that the observed range for each trait represents the expected variation for the crop in Brazil and a reference for future studies. However, the extrapolation of these results for different conditions, such as the incorporation of new varieties, more advanced harvest technology or planting in new environments should be done with caution.

Figure 4 shows the percentage of each Nutrition Composition trait but also allows a comparison among environments and among varieties within each trait when the dataset variability is partitioned. For example, when moisture in the whole dataset (Figure 4A) was partitioned by environments (Figure 4B), the data range slightly changed. It is clear that in five environments (Jaboticabal, Conchal, Rolândia, Taciba, and Montevideo) moisture values tended to overlap but in one single boxplot (Carpina) lower values tended to be more frequent than in other situations. For sucrose content and other traits, minor changes in boxplots can be found among environments. A second partitioning was done by varieties (Figure 4C), in which changes in boxplot ranges were fewer than in the partitioning by environments. These results indicate that small ranking changes can be observed when the dataset is partitioned by environments and varieties, validating our results presented in Table 2.

An important result was the dispersion of values among nutrition composition traits (Figure 4). Here, three groups arise, arranged according to the magnitudes of their values: (a) moisture, appearing in a higher percentage; (b) crude fiber, FDN, FDA, and sucrose appearing with medium percentage values; and (c) lipids, crude protein, and ash, with small percentages. The chemical composition of sugarcane is highly variable, depending on the climatic conditions, the physical, chemical and microbiological properties of the soil, the type of cultivation, the variety, the stage of maturation and age, among other factors. The sugarcane culm can be fractioned into water-insoluble substances—fibers (10–16%)—, and sugarcane juice. On average, 80% of the sugarcane juice consists of water (moisture), and 20% of sugars (e.g., sucrose), lipids, protein, and minerals (Lavanholi, 2010; Kim and Day, 2011; Gianotto et al., 2019).

This work provides information that could be a starting point for studies of substantial equivalence of sugarcane GMOs. The two substances from sugarcane that humans ingest, sugar and ethanol, are produced at high temperatures in the industry, and this minimizes any impact on food safety, because proteins or even nucleic acids would hardly be found in the final product (Joyce et al., 2013). As a smaller-scale example, the new sugarcane GM cultivar CTC91087-6, which expresses the protein Cry1Ac, protecting the plant against the sugarcane borer *(Diatraea saccharalis)*, is substantially equivalent to its conventional counterpart, and its ingestion presents minimal risks to human and animal health (Gianotto et al., 2019).

## Conclusions

The three native wild species of *Saccharum* and the plantations of sugarcane are partially sympatric in Brazil, but the likelihood of introgression is attenuated by their geographical distribution and the reproductive system of the three wild species, which prevents crossing and favors the early formation of seeds still within the rolled flag leaf.

The comparison among the chloroplast genomes provided an important framework for the comprehension of the phylogeny and the evolutionary history of the “*Saccharum* broad sense,” where the Brazilian species (*S. angustifolium, S. asperum*, and *S. villosum*) form a robust monophyletic group, together with *S. officinarum* and the commercial hybrids of sugarcane, but are less closely related to *S. arundinaceus* and *S. spontaneum*.

The nutritional composition studies revealed much genetic variation and plastic responses, and many cases of genotype-by-environment interaction. Thus, there are different responses when a given cultivar is subjected to different production environments and crop years, and the response shapes are different among the cultivars. The information generated will be included in a publicly available database (International Life Sciences Institute—ILSI) to be used in future substantial equivalence studies for genetically modified cultivars.

The three combined results generated indicate that the release of transgenic sugarcane cultivars on Brazilian territory points to no likelihood of gene transfer between sugarcane and its closest wild relatives. In addition, the nutritional composition data related to the 20 top Brazilian sugarcane cultivars are now available for future comparisons.

## Data Availability Statement

The datasets presented in this study can be found in online repositories. The names of the repository/repositories and accession number(s) can be found in the article/Supplementary Material.

## Author Contributions

EB, MB, ES, and GO conceived of the presented idea. EB, VA, RP, and AF developed the theory to study the chloroplast genome and reconstructed it. EB, GO, MB, MC, ES, and RG were responsible for the nutritional equivalence studies. EB, GO, RS, and IC were responsible for mapping the native *Saccharum* species from Brazil. All authors discussed the results and contributed to the final manuscript.

## Conflict of Interest

The authors declare that the research was conducted in the absence of any commercial or financial relationships that could be construed as a potential conflict of interest.
